# Whole genome sequencing yields the complete mitogenome of *Pandalus borealis*, an indicator species of the Arctic ecosystem

**DOI:** 10.1080/23802359.2018.1493364

**Published:** 2018-09-10

**Authors:** Gui-Cai Xu, Jun Xiao, Xiao-Qing Sun, Shang-Qi Li, Jiong-Tang Li

**Affiliations:** a Fisheries College, Zhejiang Ocean University, Zhoushan, Zhejiang, China;; b College of Fisheries and Life Science, Shanghai Ocean University, Shanghai, China;; c Key Laboratory of Aquatic Genomics, Ministry of Agriculture, CAFS Key Laboratory of Aquatic Genomics and Beijing Key Laboratory of Fishery Biotechnology, Chinese Academy of Fishery Sciences, Beijing, China

**Keywords:** *Pandalus borealis*, mitochondrial genome, phylogenetic analyses, Arctic

## Abstract

*Pandalus borealis* is an important indicator species to study the state of the Arctic ecosystem. The mitochondrial genome of *P. borealis* is 15,956 bp in length and encodes 13 protein-coding genes. The phylogenetic tree of eleven shrimps revealed that *P. borealis* belonged to *Pandalidae* family and was closely related to *C. crassicornis.* This mitogenome will be of significance to study the Arctic ecosystem state and perform the resource protection of this species.

*Pandalus borealis* is the most abundant cold-water shrimp around the Arctic (Jiao et al. 2015). Besides the commercial value, it is an important indicator to study the state of the Arctic ecosystem (Anderson 2000). Temperature, acidification, salinity, and fishing impact the global catch of this species (Bergstrom 2000; Bechmann et al. 2011). The catch has decreased from 446,909 tons (2004) to 261,435 tons (2014; http://www.fao.org/fishery/species/3425/en), suggesting that the climatic change and fishing might influence the genetic resource of *P. borealis*. Herein, we characterized the mitochondrial genome of *P. borealis* (GenBank Accession: LC341266.1).

The captive specimen and the extracted DNA samples were deposited in our laboratory (Centre for Applied Aquatic Genomics, Chinese Academy of Fishery Sciences). The genomic DNA was used to construct one paired-end library. The library was sequenced on the Illumina platform with 150 PE mode. After trimmed using SolexaQA (Cox et al. 2010), the cleaned reads (NCBI Sequence Read Archive Accession: SRX3443045) were assembled to contigs using SOAPdenovo (Luo et al. 2012). The contigs homologous to the mitochondria genomes of close shrimp were scaffolded into one long sequence using SSPACE (Boetzer et al. 2011). The gaps were filled with Gapcloser (Luo et al. 2012).

The mitogenome is 15,956 bp in length and exhibits a high A + T content of 65.57%. Thirteen protein-coding genes (PCGs), 22 tRNA genes, and two rRNA genes are identified using MITOS (Bernt et al. 2013). Six PCGs (*COX2*, *ATP6*, *COX3*, *NAD4*, *NAD4L*, and *COB*) initiate with ATG and four PCGs (*COX1*, *ATP8*, *NAD5*, and *NAD6*) with ATC. The rest PCGs use ATA and ATT as the start codons. Eleven PCGs use TAA as the stop codon and the rest two PCGs (*COX2* and *COB*) end with the incomplete stop codon T (Boore 1999).

A phylogenetic tree was constructed using Maximum likelihood (ML) analysis in MEGA package (Kumar et al. 2016) under the Jones-Taylor-Thornton model with 1000 bootstrap replicates. This tree was based on 13 mitochondrial PCGs of eleven shrimps and *Pseudocarcinus gigas* (as the outgroup). *P. borealis* was grouped with *Chlorotocus crassicornis,* both of which belong to *Pandalidae* family (Figure 1). Because of little genetic information for this family, this mitogenome will provide basic genetic data for phylogeny study. Furthermore, the result is of significance to dynamically study the Arctic ecosystem state.

**Figure 1.**
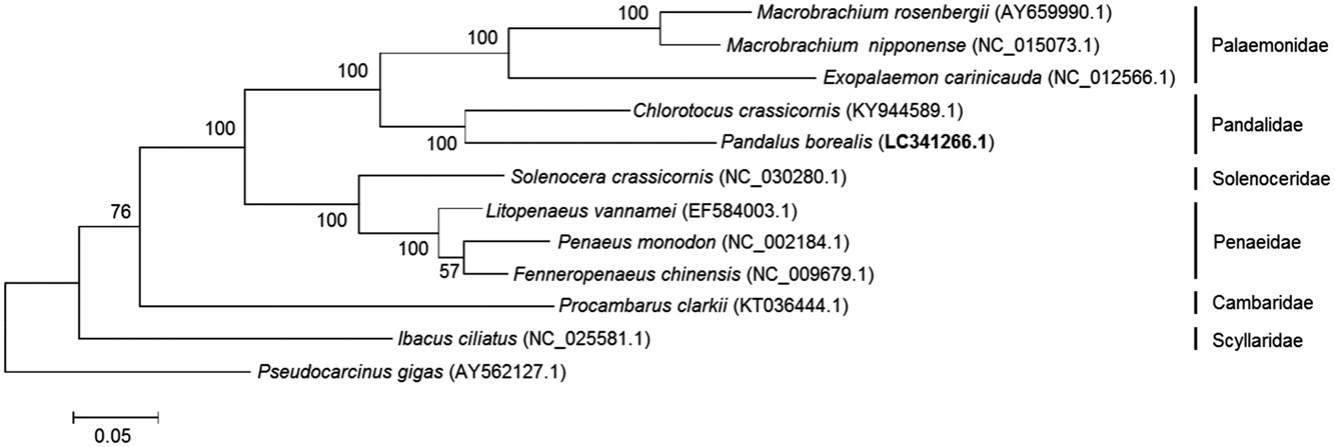
Phylogenetic tree of 12 species based on 13 mitochondrial PCGs. The number at each node is the bootstrap value of ML analysis.
